# Kinetics of Polycycloaddition of Flexible α-Azide-ω-Alkynes Having Different Spacer Length

**DOI:** 10.3390/polym15143109

**Published:** 2023-07-21

**Authors:** Andrey Galukhin, Roman Aleshin, Roman Nosov, Sergey Vyazovkin

**Affiliations:** 1 Alexander Butlerov Institute of Chemistry, Kazan Federal University, 18 Kremlevskaya Street, 420008 Kazan, Russia; 2 Department of Chemistry, University of Alabama at Birmingham, 901 S. 14th Street, Birmingham, AL 35294, USA

**Keywords:** α-azide-ω-alkynes, metal-free polycycloaddition, azide-alkyne cycloaddition, isoconversional analysis, differential scanning calorimetry

## Abstract

Two flexible α-azide-ω-alkynes differing in the length of the hydrocarbon spacers (C_8_ vs. C_12_) between functional groups are synthesized. Their bulk polymerization kinetics is studied by differential scanning calorimetry (DSC) and parameterized with the aid of isoconversional methodology. The monomer with a shorter hydrocarbon spacer has somewhat greater reactivity. The effect is traced to a moderate increase in the effective value of the preexponential factor that arises from the fact that the respective monomer has a higher initial molar concentration in itself. The techniques of GPC and NMR provide additional kinetic and mechanistic insights into the studied reaction.

## 1. Introduction

Azide-alkyne polycycloaddition (AAPC) is used widely for the creation of poly-1,2,3-triazoles [1,2,3]. In terms of the functionality, the presence of 1,2,3-triazolic units in the polymer chain enhances antifungal/antibacterial properties of polymeric materials [4], increases their flame retardation [5,6] and anticorrosion performance [6]. Metal-free (non-catalytic) AAPC is a preferable way for the synthesis of poly-1,2,3-triazoles, which are intended to be used in biological, medical or optoelectronic applications [7].

Non-catalytic AAPC is not regioselective, therefore the polymer chains formed contain a stochastic sequence of 1,4- and 1,5-disubstituited triazoles. Their ratio in the polymer chain depends on the reagents structure, the nature of a solvent and catalyst, and also affects the final material properties [7,8]. There exist two general strategies for the creation of poly-1,2,3-triazoles by means of AAPC. The first one includes reaction between two types of monomers containing alkyne and azide functionalities, respectively (A-A/B-B strategy) [1,9]. The second strategy includes application of a single monomer containing both functionalities in the same molecule (A-B strategy) [10,11]. The advantage of the second approach is that it employs a single-component reactant that secures exact stoichiometry of reacting groups, which, theoretically, should result is a higher degree of polymerization as compared to the A-A/B-B strategy [12].

The A-B strategy was implemented for both rigid (aromatic) and flexible (aliphatic) monomers containing reacting groups on the opposite sides of the monomer molecule (so called, α-azide-ω-alkynes) [10,12,13]. The polymerization of the flexible α-azide-ω-alkynes is more complex compared to that of the rigid ones because conformational lability of the former permits both inter- and intramolecular reactions [10]. The amount of cyclization products strongly depends on the concentration of the monomer in a solution and varies from few to tens of percent [10]. For rigid monomers the kinetics of polymerization was studied only briefly [13], whereas the reactivity of the flexible α-azide-ω-alkynes remains completely unexplored.

The present study focuses on the bulk polymerization of two flexible α-azide-ω-alkynes. The kinetics of the polymerization has been studied by differential scanning calorimetry (DSC) and parameterized with the aid of isoconversional methodology [14]. The structures of the studied monomers were chosen to probe the effect of the length of the hydrocarbon linker between the functional groups on their reactivity. We hypothesize that the monomer with a shorter spacer has to be more reactive because it has a higher molar concentration of the polymerizable groups. We also provide a convenient way to quantify such structure-reactivity relationship. At the same time, the ratio of carbon to nitrogen atoms in studied monomers was kept higher than three so they could be handled without a risk of explosion in a wide range of temperatures [15].

## 2. Materials and Methods

### 2.1. Materials

**Figure 1:**
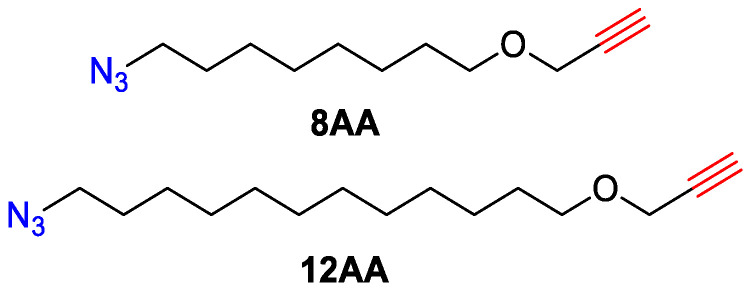
Structures of studied α-azide-ω-alkynes.

Acetonitrile (HPLC grade, ITW Reagents, Monza, Italy), 8-bromo-1-octanol (>98%, TCI, Tokyo, Japan), 12-bromo-1-dodecanol (>98%, TCI, Tokyo, Japan), NaH (>90%, Sigma Aldrich, Saint Louis, MI, USA), diethyl ether (>99%, Chimmed, Moscow, Russia), dichloromethane (>99%, Khimprom-M, Yaroslavl, Russia), N,N-dimethyl formamide (DMF, >99%, EKOS-1, Moscow, Russia), propargyl bromide (80% wt., toluene solution, Merck, Rahway, NJ, USA); silica gel (SiO_2_, 60 Å, 0.04–0.063 mm, Merck, Rahway, NJ, USA), sodium azide (>99%, Corvine Chemicals and Pharmaceuticals, Hyderabad, India), sodium sulfate (anhydrous, >99%, Khimprom-M, Yaroslavl, Russia) were purchased and used as received. Tetrahydrofuran (>99.5%, Chimmed, Moscow, Russia) was additionally distilled over sodium hydroxide. α-azide-ω-alkynes (Figure 1) have been synthesized according to the known synthetic protocol [10]. The purity of synthesized monomers was estimated from respective ^1^H NMR spectra and found to be not less than 98%.

**1-azido-8-(prop-2-yn-1-yloxy)octane (8AA)**. ^1^H NMR (400 MHz, CHCl_3_-d1, δ, ppm): 1.33 (m, 8H, CH_2_), 1.59 (m, 4H, CH_2_), 2.41 (t, 1H, J = 2.3 Hz, ≡CH), 3.25 (t, 2H, J = 6.9 Hz, CH_2_-N), 3.51 (t, 2H, J = 6.6 Hz, CH_2_-CH_2_-O), 4.13 (d, 2H, J = 2.3 Hz, O-CH_2_-C≡). ^13^C NMR (101 MHz, CHCl_3_-d1, δ, ppm): 26.13, 26.78, 28.95, 29.20, 29.38, 29.59, 51.61, 58.17, 70.34, 74.19, 80.17. IR (cm^−1^): 3304 (≡CH, ν), 2933, 2858, 2095 (N_3_, ν), 1464, 1354, 1264 (C-N, ν), 1101 (C-O-C, ν) (Figures S1–S3 in the Supplementary Materials).

**1-azido-12-(prop-2-yn-1-yloxy)dodecane (12AA)**. ^1^H NMR (400 MHz, CHCl_3_-d1, δ, ppm): 1.27 (m, 16H, CH_2_), 1.59 (m, 4H, CH_2_), 2.41 (t, 1H, J = 2.3 Hz, ≡CH), 3.25 (t, 2H, J = 7.0 Hz, CH_2_-N), 3.50 (t, 2H, J = 6.6 Hz, -CH_2_-CH_2_-O-), 4.13 (d, 2H, J = 2.3 Hz, O-CH_2_-C≡). ^13^C NMR (101 MHz, CHCl_3_-d1, δ, ppm): 26.23, 26.86, 28.98, 29.29, 29.56, 29.60, 29.65, 29.67, 51.64, 58.15, 70.46, 74.15, 80.22. IR (cm^−1^): 3308 (≡CH, ν), 2927, 2855, 2095 (N_3_, ν), 1466, 1354, 1261 (C-N, ν), 1102 (C-O-C, ν). (Figures S4–S6 in the Supplementary Materials).

### 2.2. Methods

The gel permeation chromatography (GPC) analyses were carried out with a Dionex UltiMate 3000 (Thermo Fisher Scientific, Waltham, MA, USA) chromatograph equipped with a refractive index detector RefractoMax 520 and PLgel Agilent Mixed-D column. Measurements were performed with a tetrahydrofuran (THF) as an eluent at a flow rate of 1 mL min^−1^. Polystyrene standards were used for the GPC calibration. The ^1^H and ^13^C NMR experiments were carried out on a Bruker AVANCE III NMR spectrometer operating at 400 MHz with CDCl_3_ as a solvent. ^1^H NMR analyses of the polymers were performed on the samples with incomplete conversion (~90%) for their better solubility in deuterated chloroform (Figures S7 and S8 in the Supplementary Materials). Chemical shifts are reported in delta (δ) units in parts per million (ppm). IR spectra were recorded on a Bruker Vertex 70 FTIR spectrometer.

DSC measurements were conducted with the aid of a heat flux DSC 3+ (Mettler-Toledo). instrument. Indium and zinc standards were employed for temperature, heat flow, and tau-lag calibrations. The experiments were run under 80 mL min^−1^ argon flow. Temperature was raised from 25 to 250 °C at the heating rates of 0.5, 1.0, 2.0, and 4.0 °C min^−1^. The samples were placed into 40 µL Al pans and sealed under argon. The sample mass in all runs was 5.0 ± 0.2 mg. Isothermal polymerization (130 °C, 2 h) of both monomers has been performed inside the same DSC instrument in 40 µL aluminum pans hermetically sealed in argon.

## 3. Calculations

Isoconversional kinetic analysis was conducted in accord with the recommendations of the ICTAC Kinetics Committee [14]. The ratio of the partial to total DSC peak area was used to evaluate the extents of the monomer conversion (*α*). The flexible integral isoconversional method of Vyazovkin [16] was utilized to determine the isoconversional values of the activation energy, *E_α_*. Unlike simpler rigid methods, this method allows one to eliminate a systematic error in *E_α_* incurred when *E_α_* varies considerably with *α* [16]. The elimination of the error is accomplished via piecewise integration that assumes *E_α_* to be constant only over a narrow integration range, Δ*α*. In the present computations Δ*α* was kept to be 0.01. *E_α_* was determined as the value that secures the minimum of the function:

(1) $$\Psi \left(E_{\alpha }\right)=\sum_{i=1}^{p}\sum_{j\neq i}^{p}\frac{J\left[E_{\alpha },T_{i}\left(t_{\alpha }\right)\right]}{J\left[E_{\alpha },T_{j}\left(t_{\alpha }\right)\right]}$$

where

(2) $$J\left[E_{\alpha },T_{i}\left(t_{\alpha }\right)\right]=\int_{t_{\alpha -∆\alpha }}^{t_{\alpha }}\exp\left[\frac{-E_{\alpha }}{RT_{i}\left(t\right)}\right]dt$$

and *p* is the number of the temperature programs, *T*(*t*), and *R* is the gas constant. Minimization was repeated to multiple values of *α* ranging from 0.01 to 0.99 to yield the dependence of *E_α_* on *α*. The trapezoidal rule was applied to determine numerically the values of the $J\left[E_{\alpha },T_{i}\left(t_{\alpha }\right)\right]$ integrals. A minimum of Equation (1) was found by the COBYLA non-gradient method from the NLopt library. The error bars for the *E_α_* values were evaluated as described elsewhere [17].

The dependence of ln*A_α_* on *α* was obtained by plugging the *E_α_* values into the compensation effect equation

(3) $$\ln A_{\alpha }=a+bE_{\alpha }$$

The *a* and *b* parameters were estimated by first fitting the ln*A_i_* and *E_i_* pairs into Equation (3). Each of these pairs was found by inserting various reaction models, *f_i_*(*α*) into the linearized form of the rate equation:

(4) $$\ln\left(\frac{d\alpha }{dt}\right)-\ln\left[f_{i}\left(\alpha \right)\right]=\ln A_{i}-\frac{E_{i}}{RT}$$

For each *f_i_*(*α*) model, plotting the left-hand side of Equation (4) against the reciprocal temperature permits evaluating ln*A_i_* and *E_i_* from the respective intercept and slope of the straight line. As established earlier [18], the use of four models, namely the power law (P2, P3, P4) and Avrami-Erofeev (A2), suffices for accurate calculations.

The calculated values of $J\left[E_{\alpha },T_{i}\left(t_{\alpha }\right)\right]$ and *A_α_* were employed to establish the numerical form of the integral reaction model by Equation (5):

(5) $$g\left(\alpha \right)=\sum_{\alpha }A_{\alpha }\;J\left[E_{\alpha },T_{i}\left(t_{\alpha }\right)\right]$$

To convert the data from non-isothermal to isothermal form the method of isoconversional predictions [19] was employed as represented by Equation (6):

(6) $$t_{\alpha }=\sum_{\alpha }\frac{J\left[E_{\alpha },T_{i}\left(t_{\alpha }\right)\right]}{exp\left(-\frac{E_{\alpha }}{RT}\right)}$$

where *t_α_* is the time to reach the conversion *α* at isothermal temperature, *T*.

## 4. Results and Discussion

**Figure 2:**
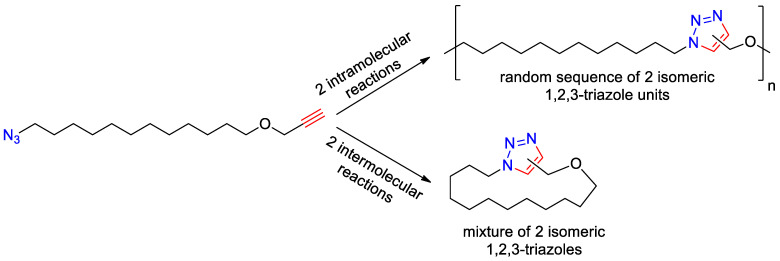
The general scheme of the studied reaction.

The studied polymerization of flexible α-azide-ω-alkynes in general includes four parallel reactions, namely intermolecular polycycloaddition and intramolecular cycloaddition, elementary act of which may result in two isomeric triazole fragments (Figure 2). Early study [10] reports that the amount of intramolecular cyclization products for flexible α-azide-ω-alkynes depends inversely on the initial concentration of the monomer in the solution and varies from 60 to 1.5%, when the concentration of the monomer changes from 0.1 to 50 wt %. This is apparently a consequence of the fact that high concentration of the monomer favors bimolecular reaction (elementary act of polymerization) rather than monomolecular cyclization. Thus, we can assume that in the bulk process, where the initial concentration of the monomer is the highest, cyclization reactions (bottom reaction path in the Figure 2) have to be suppressed, which allows us to neglect their contribution to the total process and consider only two intramolecular reactions of polymerization (top reaction path in the Figure 2). We will confirm the correctness of this assumption further.

A concerted mechanism of azide-alkyne cycloaddition assumes that the process follows the *n*th-order reaction rate law [20]. This model is commonly encountered in the kinetic analyses of azide-alkyne cycloaddition, both in solution [21,22,23] and bulk [1,9,13,24]. Since each reaction act of polymerization may result in one of the two triazolic units, reaction rate equation contains two corresponding terms:

(7) $$-\frac{dC}{dt}=\frac{dC_{1}}{dt}+\frac{dC_{2}}{dt}=k_{1}\left(T\right)C^{n}+k_{2}\left(T\right)C^{n}$$

In this equation *C*_1_ and *C*_2_ are the concentrations of isomeric triazoles, *C* is the concentration of the monomer in the reaction mixture, *n* is the reaction order, *k*_1_ and *k*_2_ are the rate constants of the formation of two isomeric 1,2,3-triazole units. The temperature dependence of the rate constant is expressed by the Arrhenius Equation (8), where the index *i* equals 1 or 2 to identify the parameters of the two respective competing reactions.

(8) $$k_{i}(T)=A_{i}\exp\left[-\frac{E_{i}}{RT}\right]$$

Considering that the concentration of the monomer relates to the extent of conversion as $C=C_{0}\left(1-\alpha \right)$ and combining Equations (6) and (7) one arrives at the following rate equation:

(9) $$\frac{d\alpha }{dt}=A_{ef,1}\exp\left[-\frac{E_{1}}{RT}\right]{\left(1-\alpha \right)}^{n}+A_{ef,2}\exp\left[-\frac{E_{2}}{RT}\right]{\left(1-\alpha \right)}^{n}$$

where

(10) $${A_{ef,i}=A_{i}C}_{0}^{n-1}$$

In the case of bulk polymerization *C*_0_ means the concentration of the monomer in itself. It should be also noted, that the *C*_0_ value in the Equation (10) is taken relative to the standard state (i.e., 1 mol L^−1^) that permits avoiding the confusion with the concentration-dependent units of the preexponential factor when the molecularity of a reaction differs from one [25].

Recently we have shown that reactions of the formation of isomeric triazoles during azide-alkyne cycloaddition of sterically unhindered reagents are characterized by the same activation energies (i.e., *E*_1_ = *E*_2_) [20]. In this case the reaction kinetics can be expressed by a simple *n*th-order reaction rate Equation (11)

(11) $$\frac{d\alpha }{dt}=A_{ef}\exp\left[-\frac{E_{1}}{RT}\right]{\left(1-\alpha \right)}^{n}$$

where

(12) $${A_{ef}=A}_{ef,1}+\;A_{ef,2}$$

**Figure 3:**
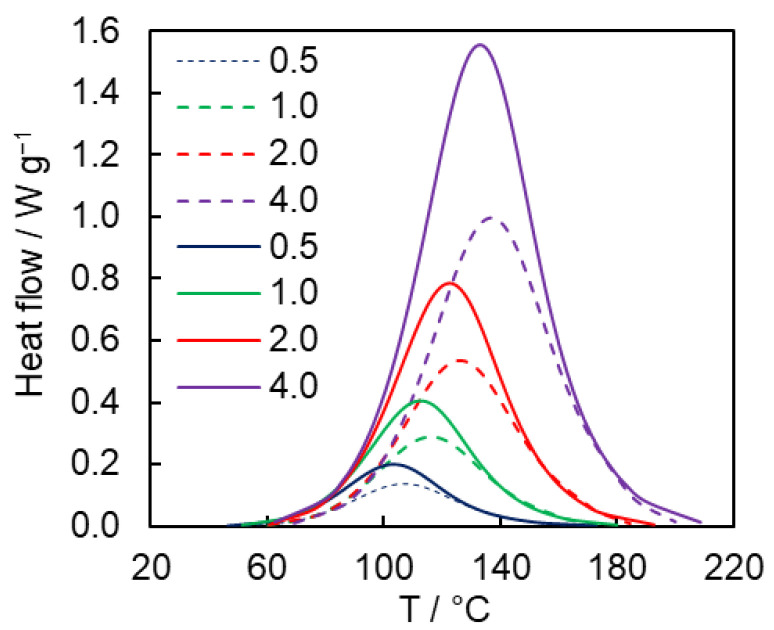
DSC curves of studied polycycloaddition reaction for **8AA** (solid lines) and **12AA** (dashed lines) monomers (numbers denote heating rates in °C min^−1^).

Since the studied reaction is highly exothermic due to the formation of aromatic triazoles, its progress can be readily followed by means of DSC. The polymerization of both monomers is conveniently studied under non-isothermal conditions. Figure 3 presents the DSC curves for the reactions under study as measured at different heating rates. Relatively slow heating rates have to be used for the DSC measurements because higher heating rates (>4 °C min^−1^) shift the reaction to the temperatures where the monomers may start to decompose [20]. As one can see from Figure 3, DSC curves for the **8AA** monomer are shifted to lower temperature compared to those for **12AA** that indicates that the former is more reactive. The difference between the corresponding peak temperatures is ~4–5 °C. The measured average heats of the reaction are 1130 ± 50 J g^−1^ (236 ± 10 kJ mol^−1^) and 790 ± 30 J g^−1^ (210 ± 8 kJ mol^−1^) for the **8AA** and **12AA** monomers, respectively. The obtained values agree with the literature data for the heats of azide-alkyne cycloaddition reactions, which usually are within the range of 210–270 kJ mol^−1^ [26].

**Figure 4:**
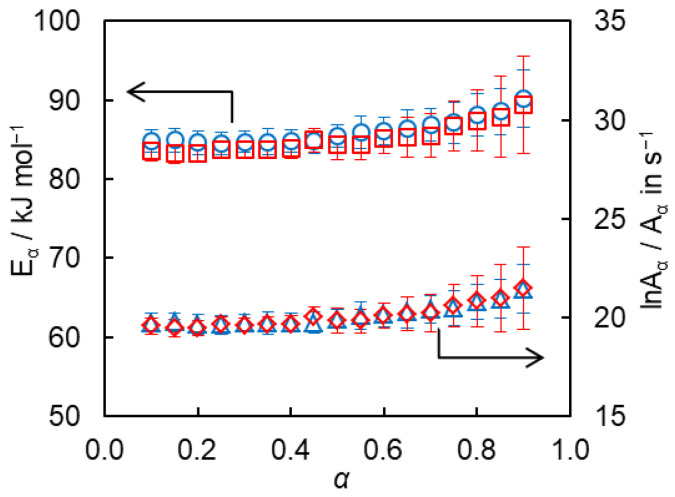
Isoconversional values of activation energy (circles and squares) and preexponential factor (triangles and diamonds) for studied for **8AA** (red symbols) and **12AA** (blue symbols) monomers.

The isoconversional kinetic analysis of the obtained calorimetric data has afforded quantification of the reactivity of the studied α-azide-ω-alkynes. Calculations yield the dependencies of the activation energy *E_α_* and pre-exponential factor *A_α_* on conversion presented in Figure 4. One can observe an insignificant variation of both parameters with conversion for the studied monomers. This suggests that the *E*_1_ and *E*_2_ values, characterizing competing steps of forming isomeric triazolic units during polymerization, are close to each other for both monomers. The average values of *E_α_* are 85 ± 2 and 86 ± 2 kJ mol^−1^ for **8AA** and **12AA**, respectively. Similar values of the activation energy are reported for other bulk AAPC reactions [13,24]. The average values of ln*A_α_* are equal to 20.0 ± 0.5 and 20.1 ± 0.4, respectively (*A_α_* in s^−1^).

**Figure 5:**
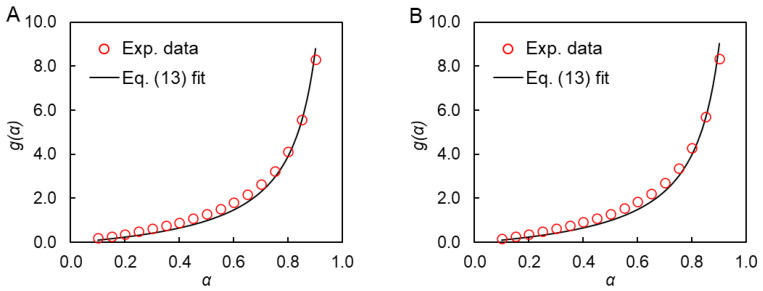
Integral form of *n*th-order reaction model fit to the experimental *g*(*α*) data for **8AA** (**A**) and **12AA** (**B**) monomers.

Substitution of the *E_α_* and *A_α_* values in Equation (5) yields numerical values of *g*(*α*) displayed in Figure 5. The dependencies of *g*(*α*) on *α* are consistent with the *n*th-order reaction model. This is readily visualized by fitting the integral form of this model (Equation (13)) to the experimental *g*(*α*) data.

(13) $$g\left(\alpha \right)=\int_{0}^{\alpha }\frac{d\alpha }{{\left(1-\alpha \right)}^{n}}=\frac{1-{\left(1-\alpha \right)}^{1-n}}{1-n}$$

The best fit *n* values and corresponding coefficients of determination (*r*^2^) values are grouped in Table 1. It is seen that the *n*th-order reaction model produces fits of good quality and yields *n* that equals 2. This value is expected naturally considering the bimolecular mechanism of the studied reaction.

Alternatively, the kinetic parameters of the polymerization have been determined by fitting Equation (11) directly to the rate data. To avoid the compensation effect between *A_ef_* and *E*_1_ the latter parameter has been fixed at the value equal to the respective mean value of *E_α_* (Figure 4). The *A_ef_* and *n* values have been optimized during fitting. The resulting kinetic parameters of the reaction are presented in Table 1. It is seen that the optimized values of the natural logarithm of the preexponential factors (Table 1) are only insignificantly smaller than the mean ln*A_α_* determined independently from the compensation effect (Figure 4). The *n* values are also smaller than those estimated via isoconversional analysis (Equation (13)), which is probably a result of mutual correlation between *n* and *A_ef_*. On the other hand, these *n* values still round off to 2.

**Figure 6:**
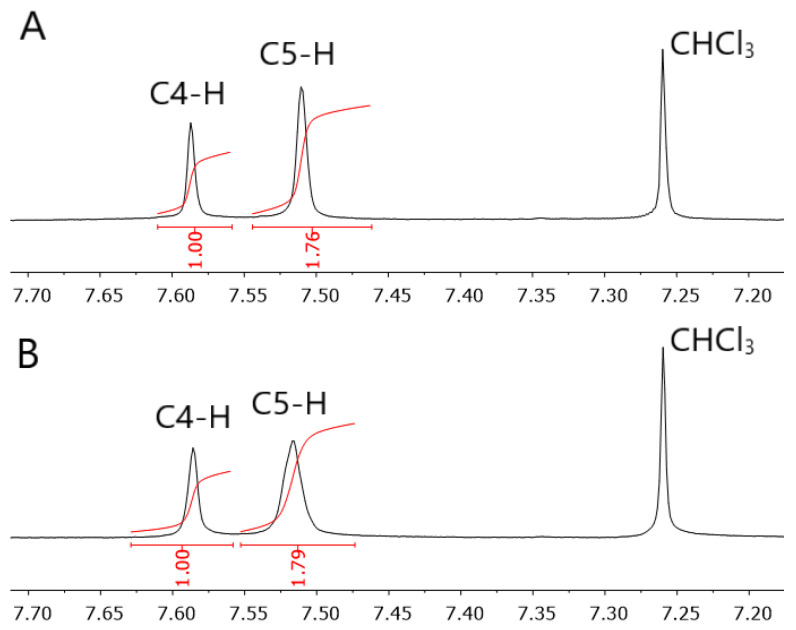
Comparison integral intensity of C4-H and C5-H protons of triazolic units in polymers based on **8AA** (**A**) and **12AA** monomers (**B**).

The obtained kinetic parameters have been used for selecting conditions suitable for completion of isothermal polymerization of the synthesized monomers. According to our calculations polymerization of both monomers at 130 °C for 2 h should result in almost complete conversion of the monomer (α > 0.98). GPC measurements of the resulting polymer samples confirm the completeness of polymerization. GPC results in the *M_n_* values of 15,600 and 17,200 g mol^−1^ for **8AA** and **12AA**, respectively. The amount of cyclization products in both cases does not exceed few percent and the contribution of intramolecular reactions can be neglected, as it was assumed previously. ^1^H NMR spectra of the obtained polymers afford estimating the ratio of 1,4- to 1,5-disubstituited triazolic fragments formed during polymerization as 1.8 in both cases (Figure 6). The same ratio of isomers has recently been reported for the bulk azide-alkyne cycloaddition between phenyl propargyl ether and 1-azidodecane [20]. It is worth noting that the ratio of the isomeric triazolic fragments in that case was found to be equal to the ratio of the preexponential factors (i.e., *A_ef,_*_1_/*A_ef,_*_2_ = 1.8) [20].

As follows from Table 1, the kinetic triplets for polymerization of both monomers are quite similar. In other words, none of the kinetic parameters reveals any obvious effect of the length of the hydrocarbon spacer on the reactivity of the studied monomers. On the other hand, it is seen experimentally (Figure 3) that the **8AA** monomer with a shorter spacer possesses somewhat higher reactivity than **12AA**. Per our hypothesis, this difference most likely relates to the higher initial molar concentration of **8AA** monomer that arises from its lower molar mass. Based on the corresponding molar masses of the **8AA** and **12AA** monomers (209.3 and 265.4 g mol^−1^) and assuming that both monomers have the same density, one can easily find that for **8AA** the *C*_0_ value in Equation (10) and, thus, *A*_*ef*,1_ should be 1.3 times higher than the corresponding values for **12AA**. Assuming for simplicity that *E* and *n* are unaffected by the spacer length, the 1.3 increase in the effective preexponential factor should cause similar increase in the polymerization rate of **8AA** relative to that of **12AA** as long as the rates are compared at the same *α* and *T* values. To perform such comparison we have calculated the isoconversional-isothermal factor *Z*_*α*,*T*_ defined by Equation (14) [27]. Isothermal polymerization rate values have been calculated from the non-isothermal ones with the help of the technique of isoconversional predictions (Equation (6)).

(14) $$Z_{\alpha ,\;T}={\left(\frac{{d\alpha }_{1}}{dt}\right)}_{\alpha ,T}/{\left(\frac{{d\alpha }_{2}}{dt}\right)}_{\alpha ,T}$$

The temperature of 130 °C has been chosen as a reference. The *Z_α,T_* value averaged over the whole conversions range has turned out to be 1.30 ± 0.04. The value of *Z_α,T_* is almost independent of the chosen temperature because of the closeness of the effective activation energies for both monomers (Table 1). Remarkably, the obtained *Z_α,T_* value matches exactly the rate increase expected from the increase in *C*_0_ and *A_ef,_*_1_ values. This apparently reinforces the idea that the effect of the hydrocarbon spacer length on the reactivity of α-azide-ω-alkynes is primarily associated with the monomer’s molar mass and its initial concentration in itself.

## 5. Conclusions

We have synthesized two flexible α-azide-ω-alkynes differing in the length of the hydrocarbon spacer between the functional groups. The kinetics of the bulk polymerization of both monomers have been studied in order to probe the effect of the spacer length. DSC measurements have detected a noticeable increase in the reactivity of the monomer with a shorter spacer length. Detailed kinetic analysis of the DSC data has revealed that both monomers demonstrate very similar overall kinetics. None of the experimentally estimated kinetic parameters could be linked specifically to the detected increase in the reactivity. However, our theoretical analysis has suggested that the increased reactivity of the monomer with a shorter hydrocarbon spacer can arise from the fact that it has a higher initial molar concentration of the functional groups. This notion has been confirmed by comparing the isoconversional-isothermal rates of polymerization of the monomers.

## Figures and Tables

**Table 1:** Estimated kinetic parameters of AAPC.

Monomer	Fit to	*E_1_*/kJ mol^−1^	ln(*A_ef_*/s^−1^)	*n*	*r^2^*
**8AA**	Equation (13)	-	-	1.99 ± 0.01	0.98
	Equation (11)	* 85 ± 2	19.67 ± 0.02	1.64 ± 0.04	0.99
**12AA**	Equation (13)	-	-	2.00 ± 0.01	0.98
	Equation (11)	* 86 ± 2	19.71 ± 0.02	1.65 ± 0.03	0.99

Symbol * denotes value kept constant during fitting.

## Data Availability

The data presented in this study are available on request from the corresponding authors.

## Supplementary Materials

The following supporting information can be downloaded at: https://www.mdpi.com/article/10.3390/polym15143109/s1, Figure S1: ^1^H NMR spectrum of 1-azido-8-(prop-2′-yn-1′-yloxy)octane (8AA) (CDCl_3_-d1, 25 °C), Figure S2: ^13^C NMR spectrum of 1-azido-8-(prop-2′-yn-1′-yloxy)octane (8AA) (CDCl_3_-d1, 25 °C), Figure S3: FTIR spectrum of 1-azido-8-(prop-2′-yn-1′-yloxy)octane (8AA), Figure S4: ^1^H NMR spectrum of 1-azido-12-(prop-2′-yn-1′-yloxy)dodecane (12AA) (CDCl_3_-d1, 25 °C), Figure S5: ^13^C NMR spectrum of 1-azido-12-(prop-2′-yn-1′-yloxy)dodecane (12AA) (CDCl_3_-d1, 25 °C), Figure S6: FTIR spectrum of 1-azido-12-(prop-2′-yn-1′-yloxy)dodecane (12AA), Figure S7: ^1^H NMR spectrum of polymer based on 1-azido-8-(prop-2′-yn-1′-yloxy)octane (8AA) (CDCl_3_-d1, 25 °C), Figure S8: ^1^H NMR spectrum of polymer based on 1-azido-12-(prop-2′-yn-1′-yloxy)dodecane (12AA) (CDCl_3_-d1, 25 °C).
