# Supplementary material for: Kinetics of Polycycloaddition of Flexible α-Azide-ω-Alkynes Having Different Spacer Length
Source: Polymers (Basel). 2023 Jul 21;15(14):3109. doi: 10.3390/polym15143109 (PMC10385033; doi:10.3390/polym15143109)
Supplement: Supplementary file 1 [file polymers-15-03109-s001.zip › polymers-2473822-supplementary.pdf]

polymers

MDPI

10.3390/polym15143109Supplementary Material: Kinetics of polycycloaddition of flexible α-azide-ω-alkynes having different spacer length

Andrey Galukhin, Roman Aleshin, Roman Nosov, Sergey Vyazovkin

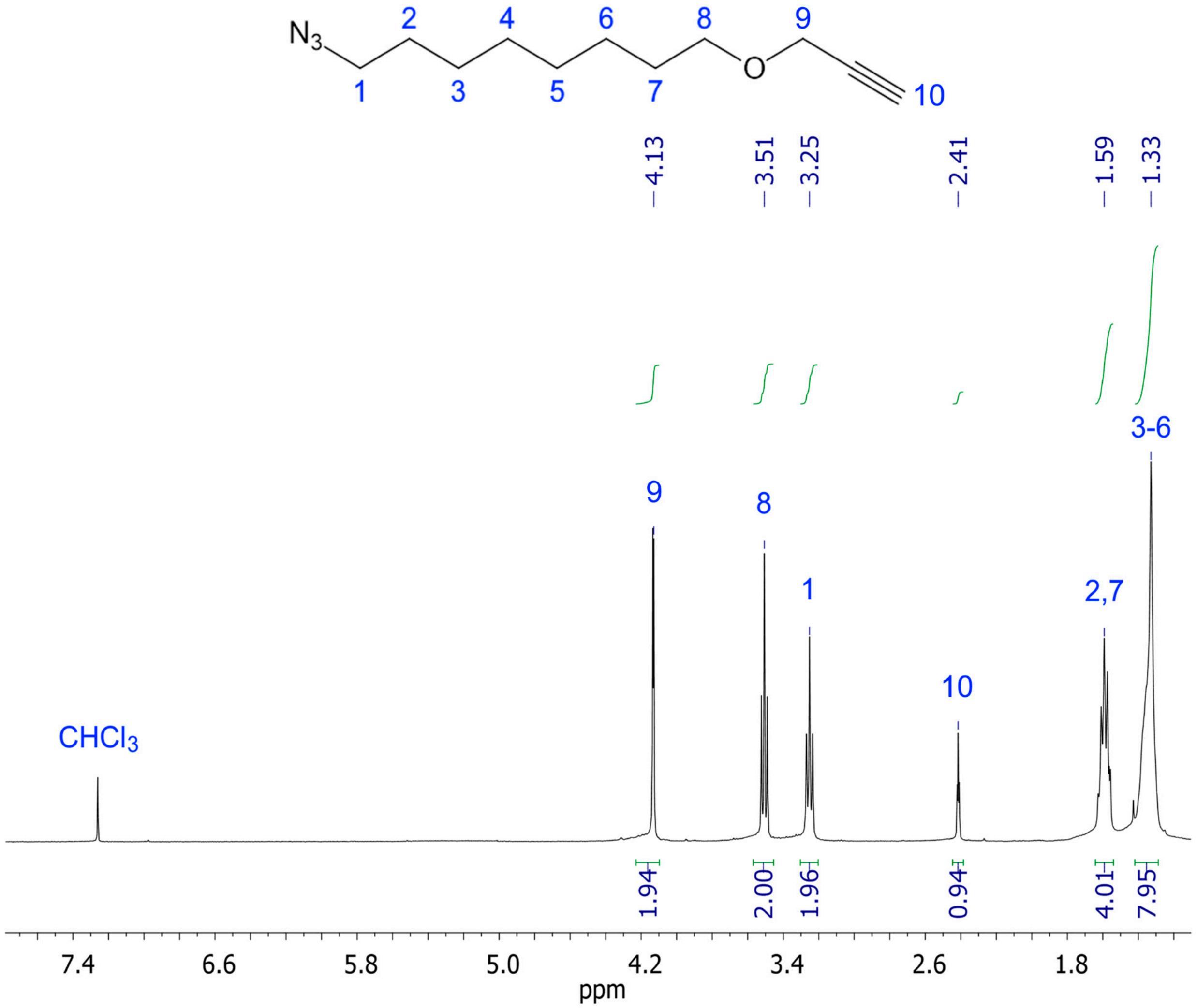

Figure S1. ^{1}H NMR spectrum of 1-azido-8-(prop-2′-yn-1′-yloxy)octane (8AA) ($CDCl_3$-d1, 25 °C).

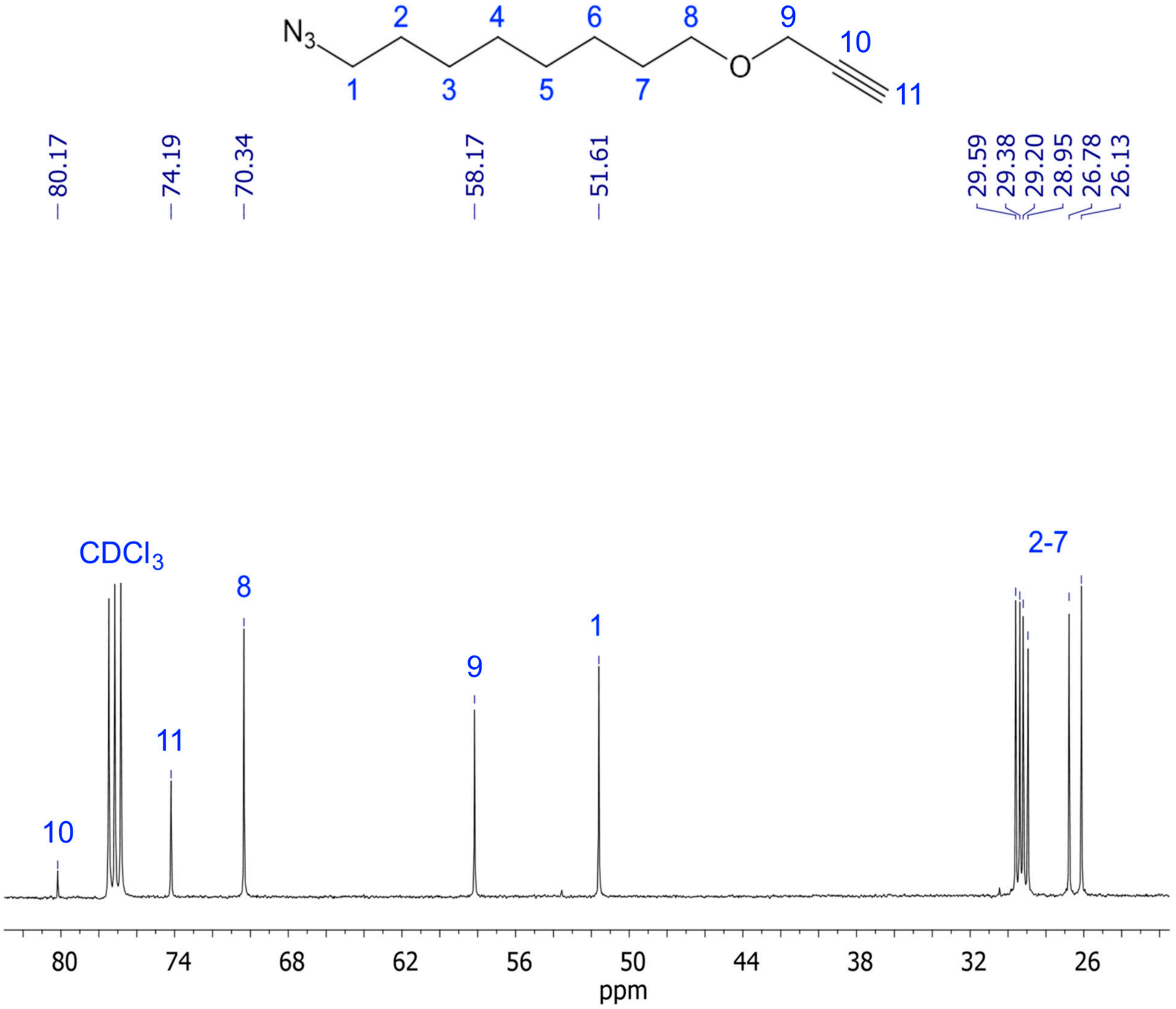

Figure S2. ^{13}C NMR spectrum of 1-azido-8-(prop-2′-yn-1′-yloxy)octane (8AA) ($CDCl_3$-d1, 25 °C).

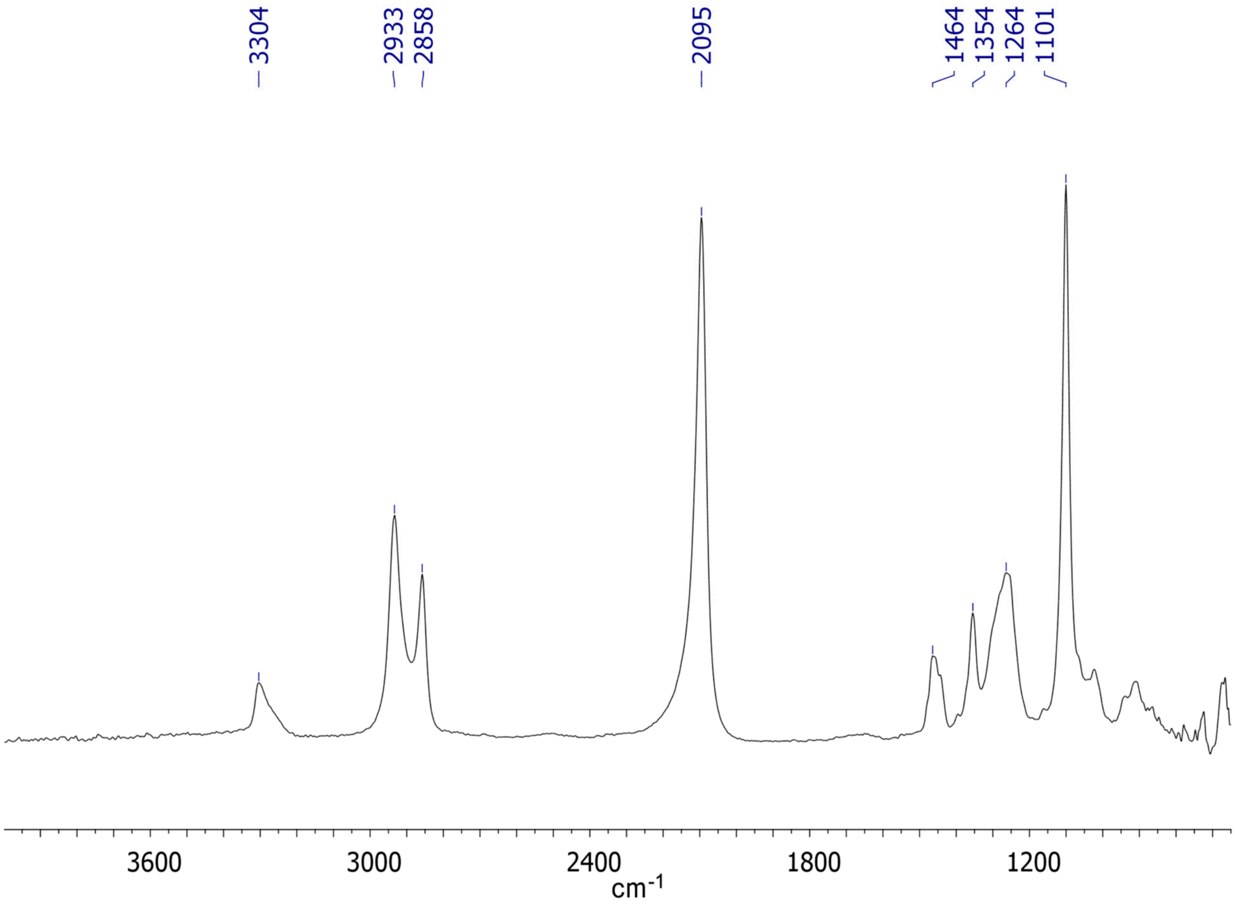

Figure S3. FTIR spectrum of 1-azido-8-(prop-2′-yn-1′-yloxy)octane (8AA).

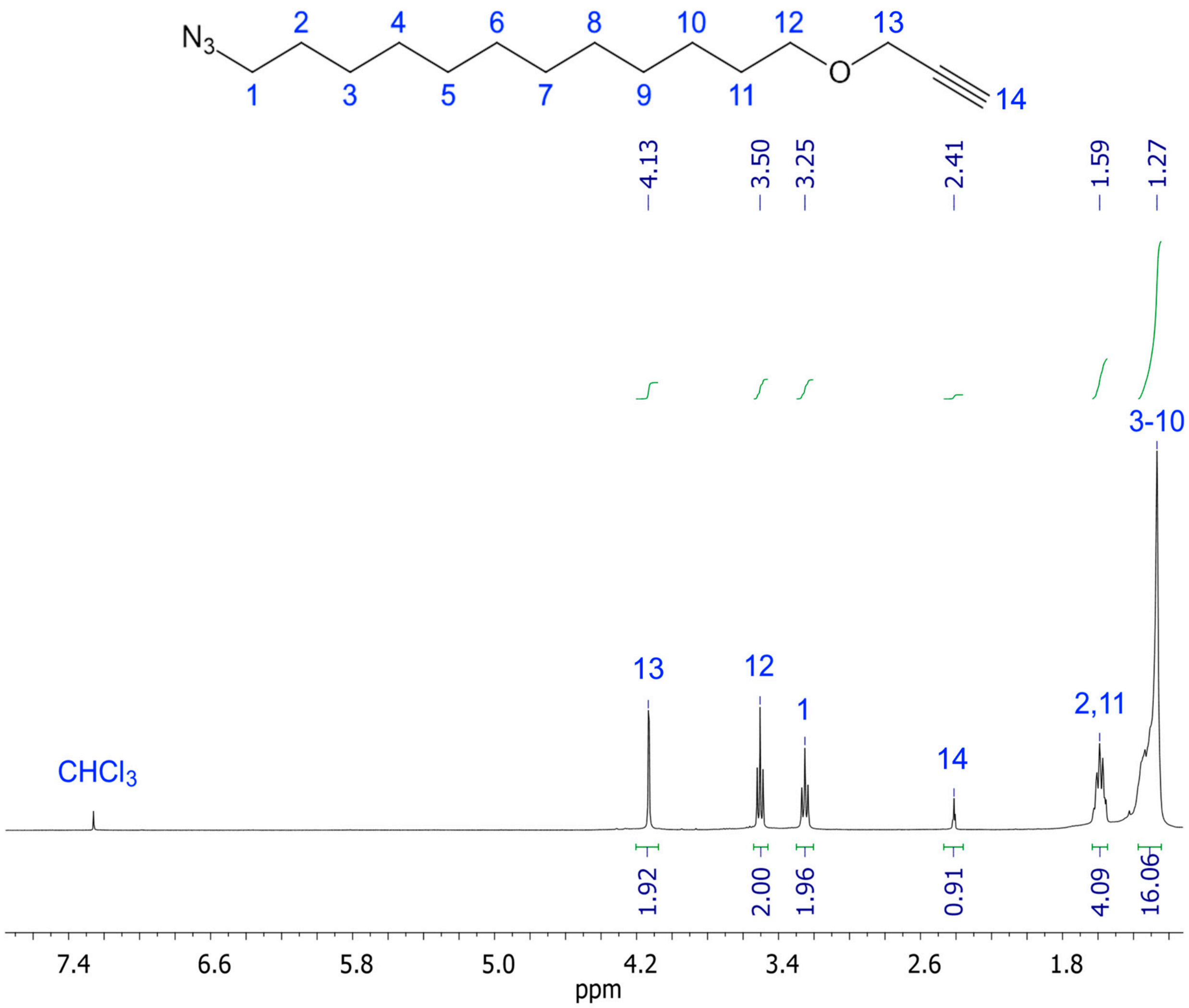

Figure S4. ^{1}H NMR spectrum of 1-azido-12-(prop-2′-yn-1′-yloxy)dodecane (12AA) ($CDCl_3$-d1, 25 °C).

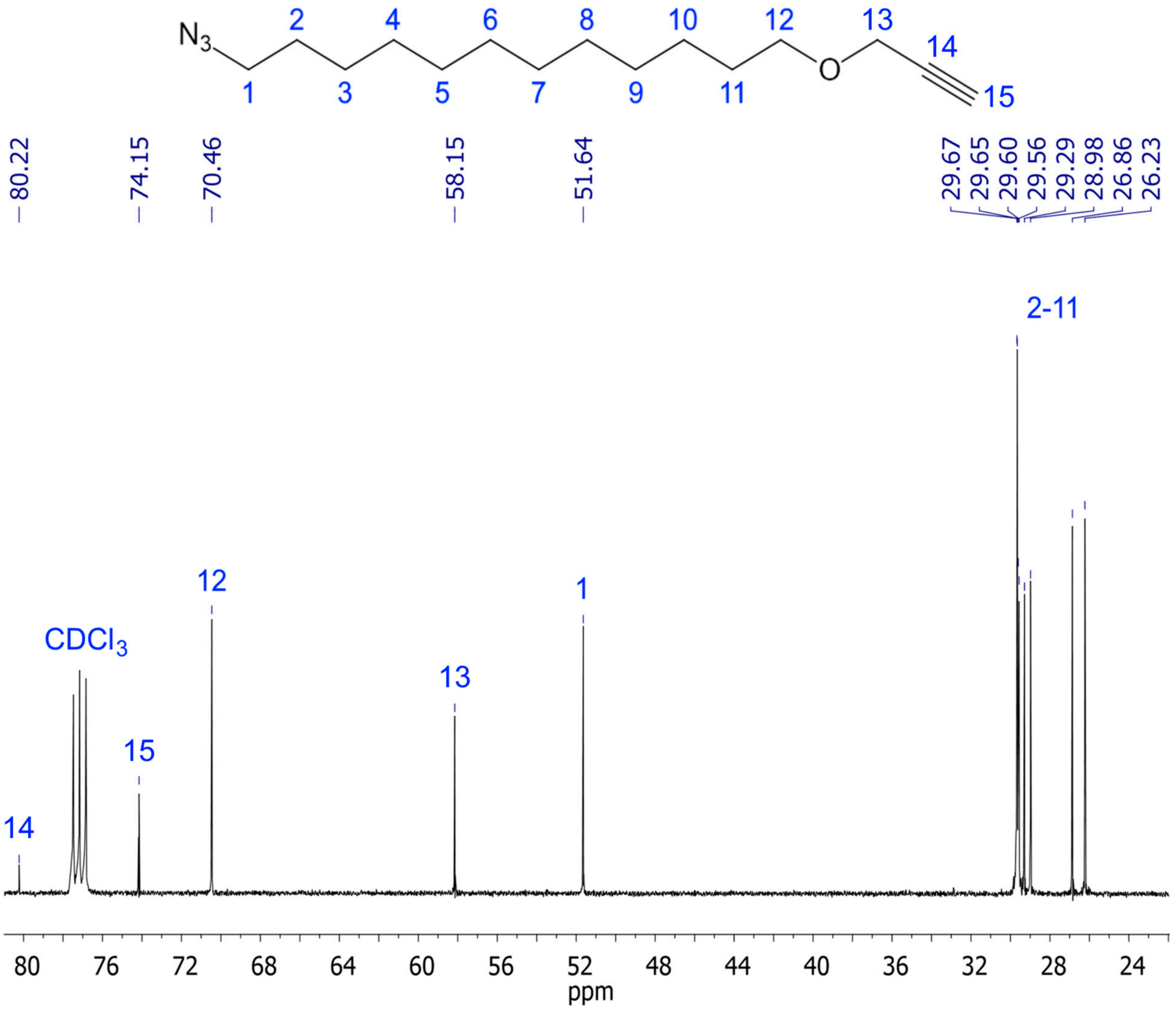

Figure S5. ^{13}C NMR spectrum of 1-azido-12-(prop-2′-yn-1′-yloxy)dodecane (12AA) ($CDCl_3$-d1, 25 °C).

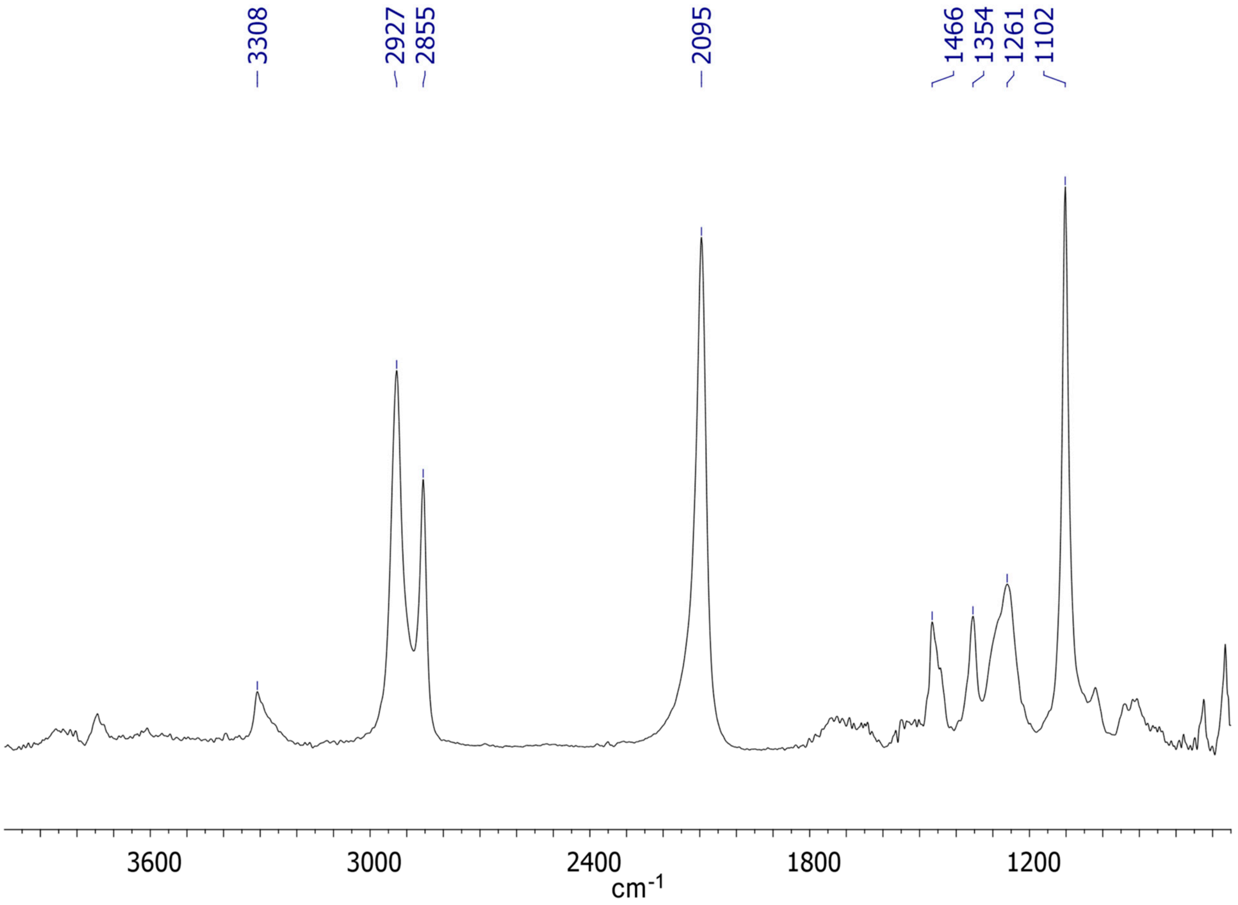

Figure S6. FTIR spectrum of 1-azido-12-(prop-2′-yn-1′-yloxy)dodecane (12AA).

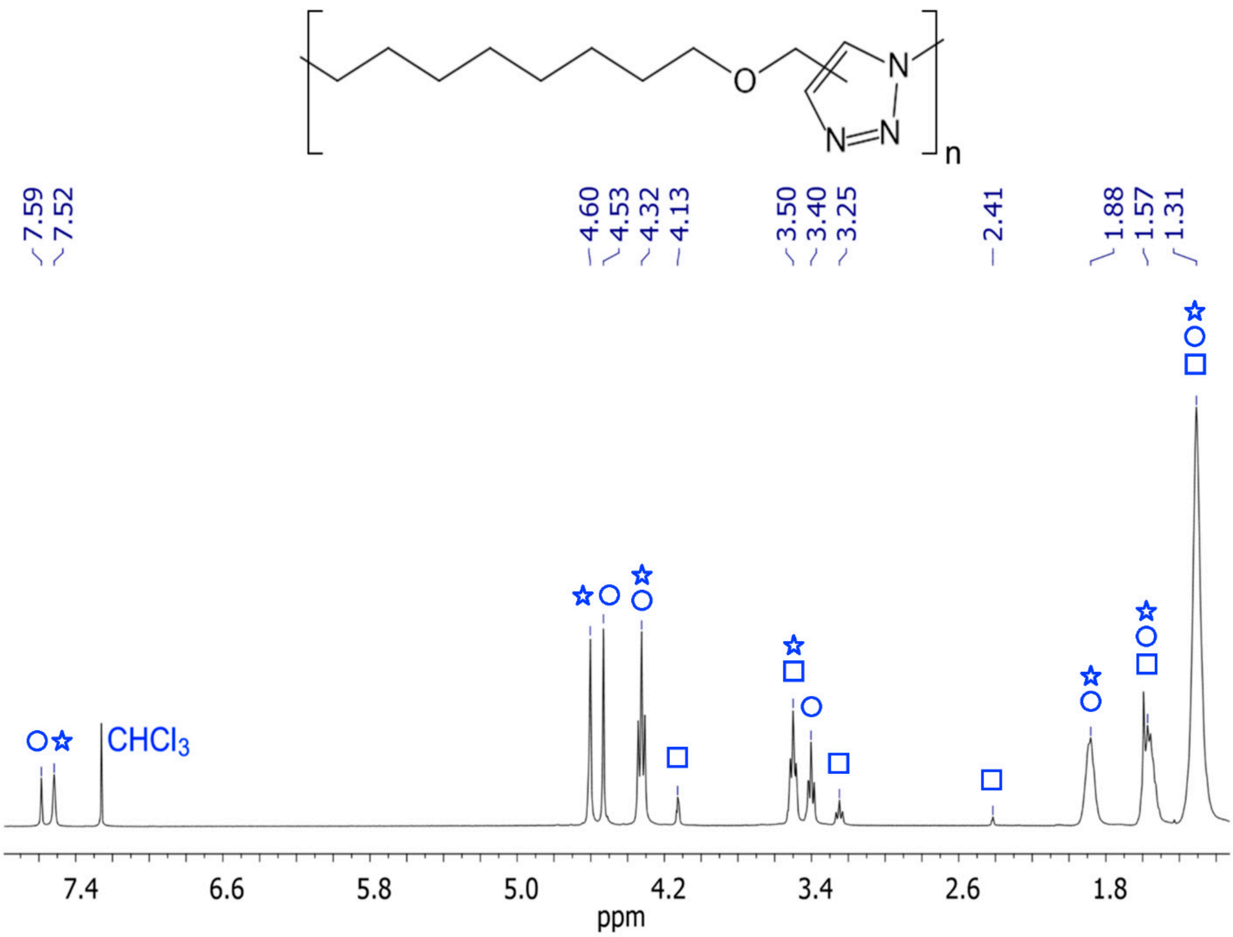

Figure S7. ^{1}H NMR spectrum of polymer based on 1-azido-8-(prop-2′-yn-1′-yloxy)octane (8AA) ($CDCl_3$-d1, 25 °C). Signals of 1,4-disubstituited triazolic unit, 1,5-disubstituited triazolic unit and of unreacted monomer are noted by stars, circles, and squares, respectively.

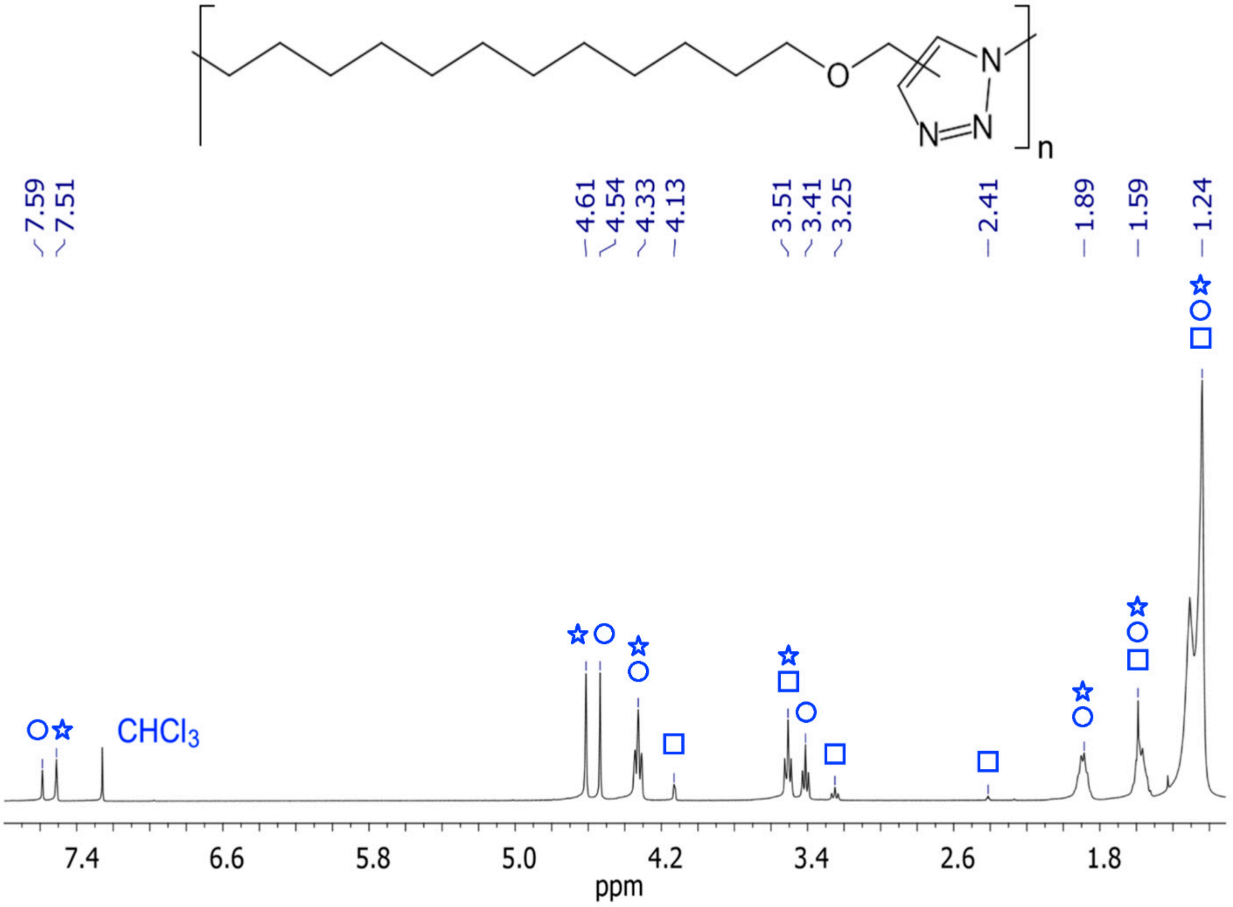

Figure S8. ^{1}H NMR spectrum of polymer based on 1-azido-12-(prop-2′-yn-1′-yloxy)dodecane (12AA) ($CDCl_3$-d1, 25 °C). Signals of 1,4-disubstituited triazolic unit, 1,5-disubstituited triazolic unit and of unreacted monomer are noted by stars, circles, and squares, respectively.
